# Nodular Hidradenoma of Left Toe

**Published:** 2015-01-22

**Authors:** Jonathan T. Nguyen, Ronald M. Brooks, Siraj M. El Jamal, Bradon J. Wilhelmi

**Affiliations:** University of Louisville Hospital, Louisville, Ky

**Keywords:** nodular, clear cell, poroid, hidradenoma, toe

## DESCRIPTION

A 26-year-old woman underwent resection of a 2-cm toe mass and adjacent skin. The defect was closed with a dorsally based rotational flap. The pathology was controversial, originally identified as digital papillary adenocarcinoma, but ultimately identified as a benign poroid/clear cell hidradenoma.

## QUESTIONS

1. **What are the histopathologic characteristics of nodular hidradenoma?**
2. **How does nodular hidradenoma present clinically?**
3. **What are competing differential diagnoses of nodular hidradenoma?**
4. **What is the management of nodular hidradenoma?**

## DISCUSSION

In 1990, Abenoza and Ackerman introduced the poroid hidradenoma (PH) as the fourth subtype of poroma. They described it as a rare, benign adnexal tumor with morphologic characteristics of both a poroma, with poroid and cuticular cells, and a hidradenoma, with both solid and cystic components confined solely within the dermis.^1^ Recently, it has been proposed to reclassify PH as a subtype of nodular hidradenoma by subdividing nodular hidradenoma into those with eccrine differentiation (PH) and those with apocrine differentiation (clear cell hidradenoma [CCH]).^2^ The pathology of nodular hidradenoma generally shows unencapsulated, eosinophilic, polygonal or fusiform cells often with sclerotic stroma, cystic spaces, and some cellular differentiation.^3^ In our patient, the pathology was atypical, with the typical features of both poroid and clear cell hidradenoma, in addition to cellular atypia and increased mitotic figures.

The clinical presentation of PH and CCH is similar. It characteristically presents as a solitary, smooth, well circumscribed, reddish to blue papule or nodule of up to a few cm in diameter.^4^ However, sizes up to 30 cm × 20 cm have been reported in literature. Patients may be asymptomatic or may present with growth, pain, or bleeding.^5^ They generally occur on the head and neck, with very rare cases on the trunk and extremities. There is a slight female predilection and age of onset is generally more than 40, though reports range from 3 to 93.^6^ Our patient was a 26-year-old woman, who presented with a growing 2-cm nodule of normal skin color on the medial aspect of the third left toe. To our knowledge, this is the first case of benign nodular hidradenoma ever to be reported in this location.

Because of the nondescript clinical presentation, the differential diagnosis is vast, including other poromas such as cylindroma or spiradenoma, other benign subcutaneous connective neoplasms such as fibroma or hemangioma, and malignant neoplasms such as papillary digital carcinoma. Diagnosis is based on histopathology, making a biopsy necessary. However, the pathology between papillary digital carcinoma and hidradenoma has significant similarities. Both tumors may exhibit papillary differentiation, mitotic figures, and atypia. It is important to differentiate them since hidradenoma is a benign lesion with extremely rare malignant transformation whereas papillary digital carcinoma is an aggressive malignant tumor that has been known to metastasize.^7^ For our patient, since acral locations are exceedingly rare for hidradenomas yet common for papillary adenocarcinomas, combined with the atypical histology, the pathology was originally read as aggressive digital papillary adenocarcinoma by one dermatopathologist, and a benign tumor by another. An expert at an outside hospital confirmed it as benign hidradenoma.

Like most other adnexal tumors, the standard of treatment is surgical resection to prevent recurrence or malignant transformation. Though rare, atypical hidradenoma does have some malignant biological potential. Wide excision and follow-up are recommended. Since hidradenomas originate from dermal tissue, simple skin sparing excision is not recommended because of risk of recurrence. Excision of the mass en bloc with overlying skin and surrounding adipose is recommended.^8^ In our patient, the tumor was isolated from neurovascular bundles and carefully removed down to the level of the phalanx. In addition, a rotational flap was performed to close the 3-cm defect. Our patient had an uncomplicated postoperative course and no evidence of recurrence at 2-month follow-up.

In summary, hidradenomas are rare, benign adnexal tumors that can easily be confused with more common malignancies such as digital papillary adenocarcinoma, but should be included in the differential diagnosis of soft tissue masses of the extremity. Given their predilection to recur and their potential to undergo malignant transformation, biopsy and surgical excision are warranted, and patients should be followed serially to monitor for recurrence.

## Figures and Tables

**Figure 1:**
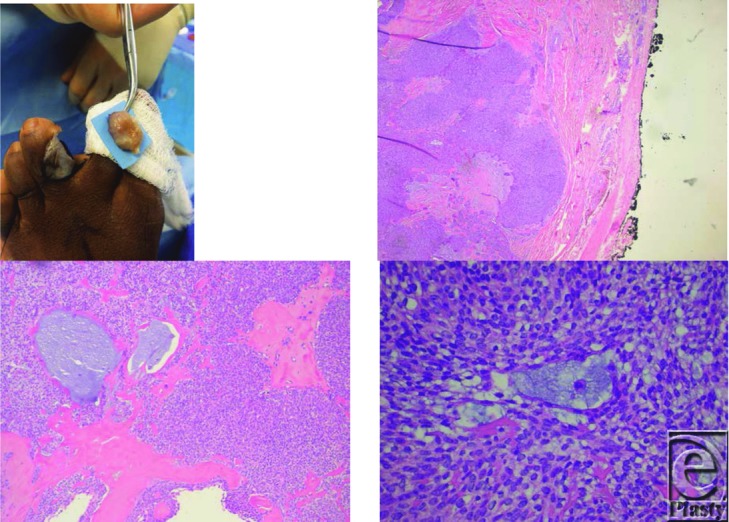
Intraoperative view of left foot showing the tumor on the third toe (*top left*). Low-powered view of mass showing well-circumscribed tumor and pseudoencapsulation (*top right*). Medium-powered view of mass showing bland morphology of cells and mucinous features (*bottom left*). High-powered view of mass showing bland morphology and mucinous features (*bottom right*).
